# Nine Months of COVID-19 Pandemic in Europe: A Comparative Time Series Analysis of Cases and Fatalities in 35 Countries

**DOI:** 10.3390/ijerph18126680

**Published:** 2021-06-21

**Authors:** David Meintrup, Martina Nowak-Machen, Stefan Borgmann

**Affiliations:** 1 Faculty of Engineering and Management, University of Applied Sciences Ingolstadt, 85049 Ingolstadt, Germany; 2 Department of Anaesthesia and Intensive Care Medicine, Ingolstadt Hospital, 85049 Ingolstadt, Germany; martina.nowak-machen@klinikum-ingolstadt.de; 3 Department of Infectious Diseases and Infection Control, Ingolstadt Hospital, 85049 Ingolstadt, Germany; stefan.borgmann@klinikum-ingolstadt.de

**Keywords:** COVID-19, SARS-CoV-2, corrected case fatality rate, time series analysis, multiple regression, flip effect, death threshold

## Abstract

(1) Background: to describe the dynamic of the pandemic across 35 European countries over a period of 9 months. (2) Methods: a three-phase time series model was fitted for 35 European countries, predicting deaths based on SARS-CoV-2 incidences. Hierarchical clustering resulted in three clusters of countries. A multiple regression model was developed predicting thresholds for COVID-19 incidences, coupled to death numbers. (3) Results: The model showed strongly connected deaths and incidences during the waves in spring and fall. The corrected case-fatality rates ranged from 2% to 20.7% in the first wave, and from 0.5% to 4.2% in the second wave. If the incidences stay below a threshold, predicted by the regression model ($R^{2}=85.0\%$), COVID-19 related deaths and incidences were not necessarily coupled. The clusters represented different regions in Europe, and the corrected case-fatality rates in each cluster flipped from high to low or vice versa. Severely and less severely affected countries flipped between the first and second wave. (4) Conclusions: COVID-19 incidences and related deaths were uncoupled during the summer but coupled during two waves. Once a country-specific threshold of infections is reached, death numbers will start to rise, allowing health care systems and countries to prepare.

## 1. Introduction

On 24 January 2020, the first three cases of COVID-19 in continental Europe were anounced in France [1]. Two months later on 17 March 2020, Montenegro was the last country in Europe to report at least one case of COVID-19. In the early phase of the pandemic, transmission rates in Europe were comparably low [2]. Nevertheless, by the end of the year 2020, more than 22 million confirmed cases and more than 500,000 COVID-19 related deaths were counted by the European health authorities. However, infection rates with SARS-CoV-2 and the related mortality of COVID-19 have not been equal among European countries. Significant differences both in infections and COVID related death rates have been observed on a local, regional and national level [3,4,5].

An uncontrolled spread of SARS-CoV-2 infections would have disastrous consequences for individuals and health care systems worldwide. However, the severe global economic and social impact of lockdown measures needs to be taken into consideration. Gaining a better understanding of dynamic patterns exhibited by the pandemic in Europe is paramount as political inequalities between countries followed by at times opposing strategies to fight the pandemic has made Europe a challenging place to contain the virus.

Observing variables in fixed time intervals, like the daily numbers of COVID-19 incidences and related deaths in each country, naturally produces time series. In the ongoing research focussing on these time series, one can distinguish three main directions: First, the temporal dynamics of COVID-19 incidences and related deaths at the onset of the pandemic in a specific country [6,7,8,9]; second, the correlation between COVID-19 incidences and another factor of interest, ranging from air pollution [10] through social capital [11] to stroke admissions [12]; third, the effect of political measures—from social distancing to complete lockdown of public life—on COVID-19 incidence and related death numbers [13,14,15].

In the present study, we sought to model the temporal relation between COVID-19 incidences and related deaths for 35 European countries with at least 1 million inhabitants, extending our analysis over a period of nine months, including both spring and fall waves of 2020 and the relatively quiet summer months of 2020 observed in most European countries.

We hypothesized that incidence rates and death rates are coupled at certain times, whereas an uncoupling of incidence rates and death rates can be observed when incidences stay below a certain infection threshold. The goal of our study was to describe the dependency of death rates from COVID-19 incidences, taking into account the time delay between infection and death, the changes in mortality over time, and the differences from country to country. Additionally, we aimed to estimate the threshold value of coupled death and incidence rates for each country. The geographical structure of the clusters can provide insights about the dynamic of the spread of the SARS-CoV-2 virus across Europe.

## 2. Materials and Methods

### 2.1. Time Series Analysis

Our analysis is based on the COVID-19 incidences and deaths data from the European Centre for Disease Prevention and Control [1]. We restricted our attention to the 35 European countries with a population of more than 1 million. In order to obtain stable results, we used the 7-day moving average of the COVID-19 incidences and of the COVID-19 related deaths.

By 30 November, the pandemic progressed in three distinct phases in most European countries. During the first and third phase, commonly referred to as first and second “waves”, the case fatality rate (CFR) was strongly connected to the incidence rate, typically with a higher percentage of COVID-19 related deaths in the first than in the second wave. In the phase in between the two waves, the CFR was independent from incidence appearing as a random variable around a constant value. In most countries, the first and third phase occurred in the spring and fall of 2020, while the second “quiet phase” was observed during the summer months of 2020 [1].

In order to validate the three different phases of the pandemic for Europe, the following time series model was used:

(1) $$y_{t}=\left\{\begin{array}{cc} \alpha_{1}\cdot x_{t-d_{1}}, & \mathrm{for}\;1\leq t\leq t_{1},\\ p_{2}, & \mathrm{for}\;t_{1}<t<t_{2},\\ \alpha_{2}\cdot x_{t-d_{2}}, & \mathrm{for}\;t\geq t_{2}. \end{array}\right.$$

We evaluated a period of nine months, beginning on 1 March 2020 (t=1), and ending on 30 November 2020 (t=275). In this model, $\left(x_{t}\right)$ is the 7-day moving average of COVID-19 incidences, and $\left(y_{t}\right)$ is the (predicted) 7-day moving average of COVID-19 related deaths on day *t*. The model parameters $\alpha_{1}$ and $\alpha_{2}$ ($0\leq \alpha_{1,2}\leq 1$, discretized with an increment of 0.001 for optimization), correspond to the percentage of incidences that led to a fatal outcome. We will refer to $\alpha_{1}$ and $\alpha_{2}$ as corrected case fatality rates (cCFR). In order to obtain proper case fatality rates, it was necessary to calculate country specific shift parameters ($d_{1}$ and $d_{2}$ in the model) to account for the correct censoring of the data [16]. The integer-valued shift parameters $d_{1}$ and $d_{2}$ represent the number of days between the date of reporting the number of COVID-19 cases and the date of reporting the number of COVID-19 associated deaths. In addition, differences in the efficiency of treatment and in the case reporting systems of the countries can affect the time between a positive test result and death, and hence impact these shift parameters.

The model was fitted individually to each country using a minimum error variance approach. The optimizer that we wrote iterated through all possible integer values for $d_{1}$, $d_{2}$, $t_{1}$, $t_{2}$ (with the constraint $t_{2}>t_{1}$) and all $0\leq \alpha_{1},\alpha_{2}\leq 1$ (with step size of 0.001) in order to find the minimum of the loss function. It is crucial to note that the optimization did not only include the cCFRs ($\alpha_{1}$,$\alpha_{2}$), the average number of deaths in phase 2 ($p_{2}$), and the shift parameters ($d_{1}$,$d_{2}$), but also the points in time $t_{1}$ and $t_{2}$, where the switch between the phases happened. Hence, in the fitting process, all subdivisions of the nine months in three phases were considered in order to find the best possible time intervals. This implies that the model-fit automatically detects the time interval in which a constant death rate, unconnected to the number of COVID-19 incidences, fits the data better than a coupled death rate proportional to the number of incidences. More precisely, a better model fit means a smaller value of the loss function, which is given by the mean squared error.

### 2.2. Clustering

The time series analysis described above provides a set of 7 parameters ($\alpha_{1}$,$\alpha_{2}$, $p_{2}$, $d_{1}$, $d_{2}$, $t_{1}$, $t_{2}$) for each country. These parameters were used to assign countries to specific groups (“clusters”) with similar epidemiology. The relative death rate per 100,000 inhabitants in the second phase was calculated for each country:

(2) $$p_{2rel}=p_{2}\cdot \frac{100,000}{\mathrm{Population}\;\mathrm{Size}}.$$

The three parameters ($\alpha_{1}$,$\alpha_{2}$, $p_{2rel}$) represent relative, and hence comparable measures of the case fatalities for the three phases in each country. As the absolute magnitude of the α-values changes significantly between the first and the second wave, we assigned each country to its corresponding α-ranks $r_{1}$ and $r_{2}$. The ranks are ascending, meaning that smaller α-values correspond to lower ranks. The rank difference

(3) $$\Delta r=r_{1}-r_{2}$$

is an expression of the change in rank of each country from the first to the second wave and reflects aspects of the dynamics of the pandemic. Hence, the three severity measures and the rank difference ($\alpha_{1}$,$\alpha_{2}$, $p_{2rel}$, Δr) were used for the cluster algorithm. The hierarchical clustering was performed with Ward’s method and standardized variables [17].

### 2.3. Regression

After a relatively quiet summer, in most countries, the COVID-19 incidences started to rise again in the fall, and, at a certain individual time point, COVID-19 related deaths started to rise and reconnected with the incidences. We hypothesize that each country has an individual threshold value $C_{T}$. Once this limit is exceeded, a re-coupling of the death rate with the incidence rate can be observed. The beginning of phase 3, the day $t_{2}$, is an optimized parameter in our time series analysis. It can be used to estimate the threshold value $C_{T}$ that leads to reconnection of the COVID-19 incidences and the related death rate in a specific country:

(4) $$C_{T}=x_{a},\;\mathrm{with}\;a=t_{2}-d_{2}.$$

The day $t_{2}$ is the point in time, where the connected model fits the data better (i.e., leading to a smaller value of the loss function) than the unconnected model. We find the corresponding threshold value of incidences by going back $d_{2}$ days, the country-specific delay between incidence and death.

It is plausible to assume that the threshold value $C_{T}$ mainly depends on the population size of each country, but also on the severity of the first and second phase, expressed in $\alpha_{1}$ and $p_{2rel}$. We therefore fitted a multiple linear regression model with $\ln(C_{T})$—the natural logarithm of the threshold value–as response variable, and ln(P)—the natural logarithm of the population size–and the parameters $\alpha_{1}$ and $p_{2rel}$ as factors:

(5) $$\ln\left(C_{T}\right)=c_{0}+c_{1}\cdot \ln\left(P\right)+c_{2}\cdot \alpha_{1}+c_{3}\cdot p_{2rel}.$$

When this linear regression model fits the data well, it can be used to predict the threshold value of COVID-19 incidences for each country that will lead to a reconnection of COVID-19 cases and related deaths.

## 3. Other Data

Information about gross domestic product (GDP) of the countries analyzed herein provided by Wikipedia [18]. Cumulative number of infected and deceased individuals in these countries was obtained from the homepage of the World Health Organization (WHO) [19] on 13 April during the third wave.

All statistical analyses were performed with the statistical software JMP${}^{®}$ Pro [20].

## 4. Results

**Figure 1:**
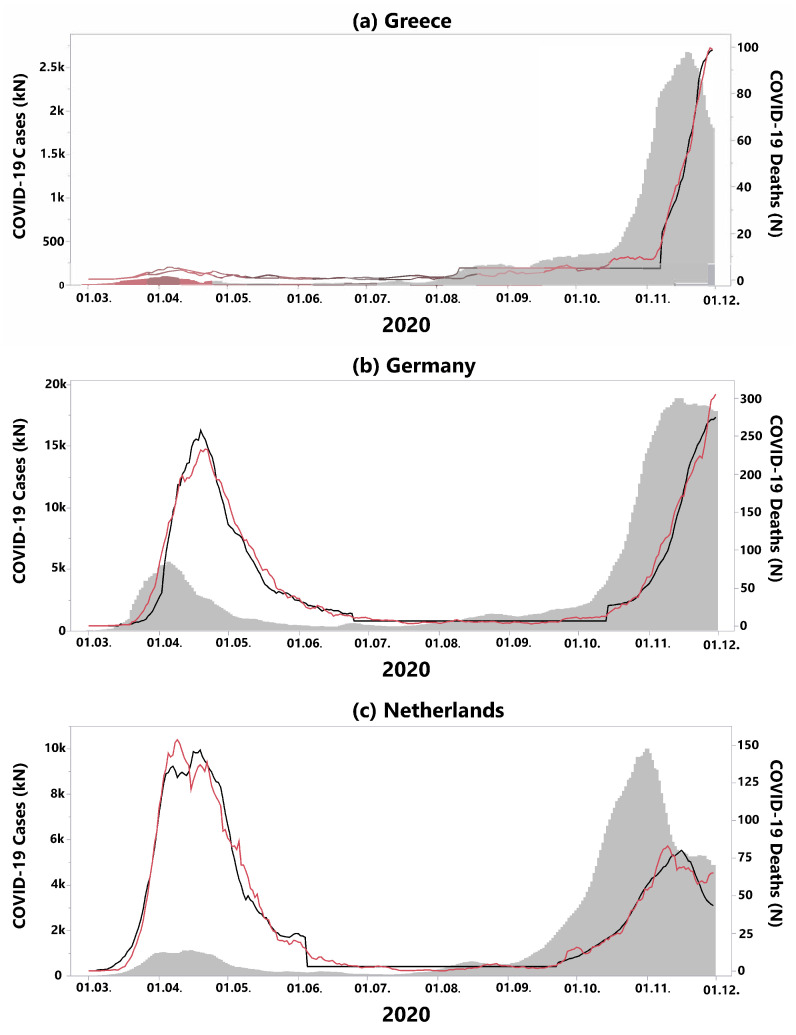
Examples of time series analyses performed on the temporal incidence of SARS-CoV-2 infections (grey area, left scale) and the incidence of COVID-19 associated deaths (red line, right scale). The three-phase-prediction model (black line, right scale) for COVID-19 associated deaths based on actual incidences has been estimated for each country. The second phase of the model with a constant number of deaths is identifiable by the horizontal black line. (**a**) Greece (cluster 1), exhibits a constant increase of incidences over time ($\alpha_{1}=4.7\%$ and $\alpha_{2}=4.2\%$). (**b**) For Germany ($\alpha_{1}=4.5\%$ and $\alpha_{2}=1.5\%$—cluster 2), and (**c**) the Netherlands ($\alpha_{1}=13.1\%$ and $\alpha_{2}=0.8\%$—cluster 3), the threshold effect is particularly visible: the case fatality rates did not increase during the summer months, despite a relevant increase of COVID-19 incidences.

Table 1 contains the results of our statistical analysis for the 35 European countries that were included in our study. Following the country name, the next seven columns contain the parameter estimates of our model (see Equation (1)). Based on these parameter estimates, countries were assigned to groups with similar outcomes (clusters). A typical representative of the time series model for each cluster is displayed in Figure 1: Greece, representing cluster 1, Germany, representing cluster 2, and the Netherlands, representing cluster 3. The table is organized by cluster, and alphabetically by country name within each of the three clusters.

**Figure 2:**
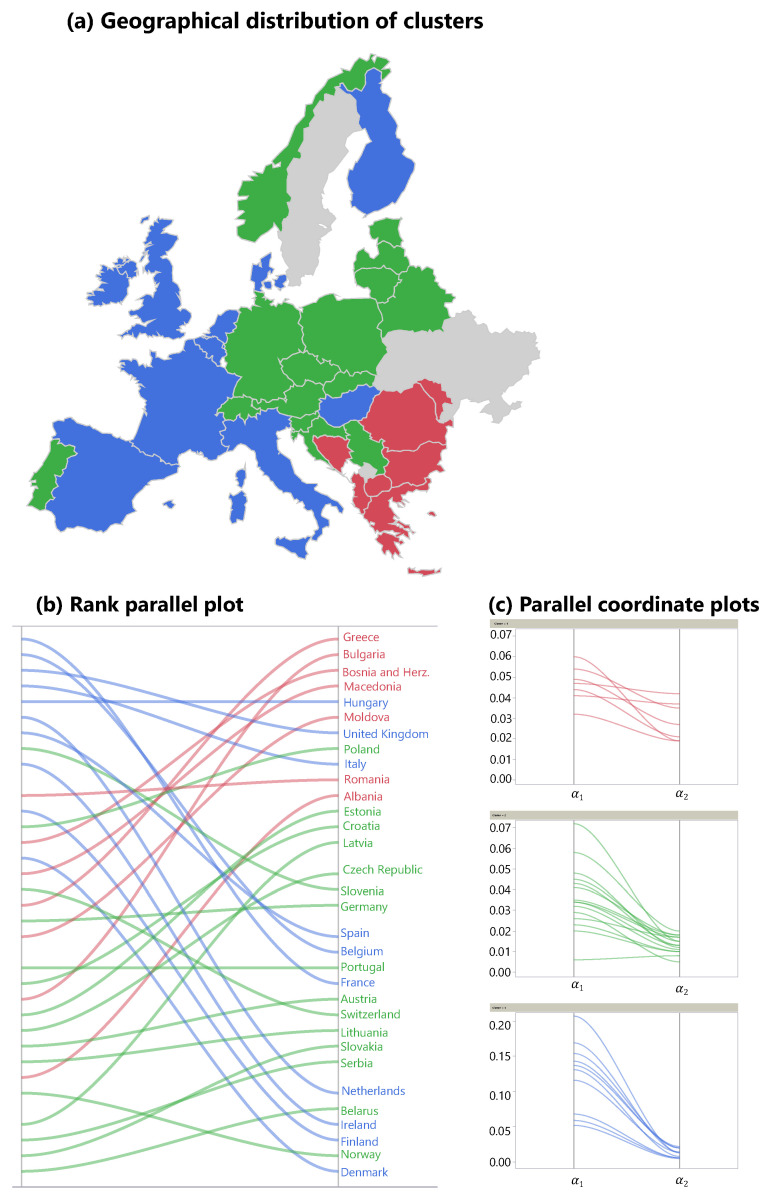
Graphical representations of the clustering. The three clusters were determined using the four parameters ($\alpha_{1}$, $\alpha_{2}$, $p_{2rel}$, Δr) deduced from the time series analysis. (**a**) Although these parameters did not contain any geographical information, the clusters represent different regions in Europe. The parallel plot of the α-ranks shows that the first cluster (red, southeast Europe) was characterized by low cCFRs in the first wave and high values in the second wave (compared to the rest of Europe). The third cluster (blue, western Europe) exhibited an opposite pattern. In the second cluster (green, central Europe), low cCFRs in the first wave were followed by slightly higher cCFRs in the second wave. In grey: countries not attributable to a particular cluster. (**b**) Overall, the rank plot shows evidence of the flip effect: in nearly all countries, the α-rank changed. (**c**) Absolute values of $\alpha_{1}$ and $\alpha_{2}$ of the three clusters. Note that these values are standardized for the clustering algorithm.

Cluster 1 consists of countries that were mildly affected by the first wave, but suffered from high cCFRs in the second wave (Figure 2a red cluster, Figure 2b rank parallel plot). In addition, these countries exhibit high relative death rates $\left(p_{2rel}\right)$ during the “quiet” phase 2. Cluster 1 can be described as countries in which the CFRs increased slowly over the course of two waves, but, unfortunately, the situation declined constantly and ultimately led to the highest cCFRs in all of Europe. Countries in cluster 1 are all geographically located in the South-East of Europe. A typical representative of cluster 1 is Greece (see Figure 1a), switching from a relatively low $\alpha_{1}=4.7\%$ to a relatively high $\alpha_{2}=4.2\%$, which was the highest value of all considered countries in the second wave.

Cluster 2 includes Central European countries such as Germany, Poland, and the Czech Republic (Figure 2a). These countries started with the lowest cCFRs across Europe in the first wave. Cluster 2 also showed a slow progression of CFRs during the pandemic; however, compared to cluster 1, the CFRs in cluster 2 never reached the same increase in CFR during the second wave. In Germany, for example (Figure 1b), during the first wave, $\alpha_{1}=4.5\%$ was similar to $\alpha_{1}=4.7\%$ of Greece. However, Germany‘s CFR during the second wave (phase 3) remained much lower than the CFR of Greece during the same time period ($\alpha_{2}=1.5\%$). Furthermore, in Germany, the incidences during the summer months (phase 2) declined substantially when compared to the first wave (phase 1) which marks a difference between the countries in cluster 1 where the incidences failed to drop during phase 2.

The third cluster, located in Western Europe (blue countries in Figure 2), showed an opposite course to cluster 1. As demonstrated in the rank parallel plot (Figure 2b), countries in cluster 3 started out with the highest cCFRs across Europe in the first wave, and switched to relatively low CFRs in the second wave. France, for example, had the highest cCFR of all 35 countries in the first wave ($\alpha_{1}=20.7\%$), while in the second wave, despite a high number of incidences, the cCFR was low ($\alpha_{2}=1.3\%$ ). In the Netherlands (cluster 3, Figure 1c), the first wave led to a very high $\alpha_{1}=13.1\%$, followed by an absolute and relative decrease to $\alpha_{2}=0.8\%$.

The 5-point summary statistics for the five model parameters ($\alpha_{1}$,$d_{1}$, $\alpha_{2}$, $d_{2}$, $p_{2rel}$) are summarized in Table 2.

In general, cCFRs in Europe were lower in the fall (phase 3, second wave) than in the spring (phase 1, first wave) of 2020. While the median of the cCFRs during the first wave was 4.8%, it dropped to 1.5% in the second wave. The shift parameters increased from nine days in the first wave to 12 days in the second wave. Both results indicate a less vulnerable population and improved treatment of COVID-19 patients during the second wave.

The fact that the constant model for the death rate was the best fit for the data in most countries during the summer months has another important consequence. In many European countries, the COVID-19 incidences were slightly increasing over the summer, whereas death rates remained low. This result undermines our findings that incidences and death rates are not necessarily connected, as long as the incidences stay below a certain threshold, which can be described as an “uncoupling” of incidences and deaths. Once an individual threshold of infections in a country is reached, the death rates will begin to increase following the incidence rates, which can be described as the “re-coupling” of incidences and deaths. This “threshold” effect is shown in Figure 1 for representative countries of all three clusters.

Table 3 shows examples of uncoupling for Austria, France, Germany, and Denmark during the quiet summer months of the pandemic, where incidences were slowly increasing as the pandemic approached the second wave. In all four countries, the number of cases increased between 2- and 4-fold. In contrast to the first (phase 1) and second wave (phase 3), the absolute number of COVID-19 related deaths stayed constantly low.

Column 9 in Table 1 contains difference in α-ranks (see Equation (3)), followed by the threshold value $C_{T}$ (see Equation (4)). Based on the relative increase of the distance metric in the hierarchical clustering, three clusters were used with 7, 10, and 15 members, respectively. The mean values of the four parameters ($\alpha_{1}$, $\alpha_{2}$, $p_{2rel}$, Δr) that were used for the cluster algorithm are displayed in Table 4.

The last column in Table 1 displays the cluster the corresponding country has been assigned to. A geographical representation of the clusters is shown in Figure 2a. The parallel coordinate plots in Figure 2c show the absolute values of the parameters ($\alpha_{1}$, $\alpha_{2}$) for each country in the three clusters. The last parameter Δr is represented in Figure 2b. Each line in this graph connects the rank of $\alpha_{1}$ on the left side with the corresponding rank of $\alpha_{2}$ on the right side for the given country. The rank difference Δr between both sides represents the relative improvement or deterioration, and was used as a fourth parameter in the clustering process.

The linear regression model (see Equation (5)) is given by Equation

(6) $$\ln\left(C_{T}\right)=-3.78+0.87\cdot \ln\left(P\right)+2.86\cdot \alpha_{1}+1.50\cdot p_{2rel}.$$

The variance inflation factors of all involved factors were below 2, so that there is no indication of multicollinearity in the data.

**Figure 3:**
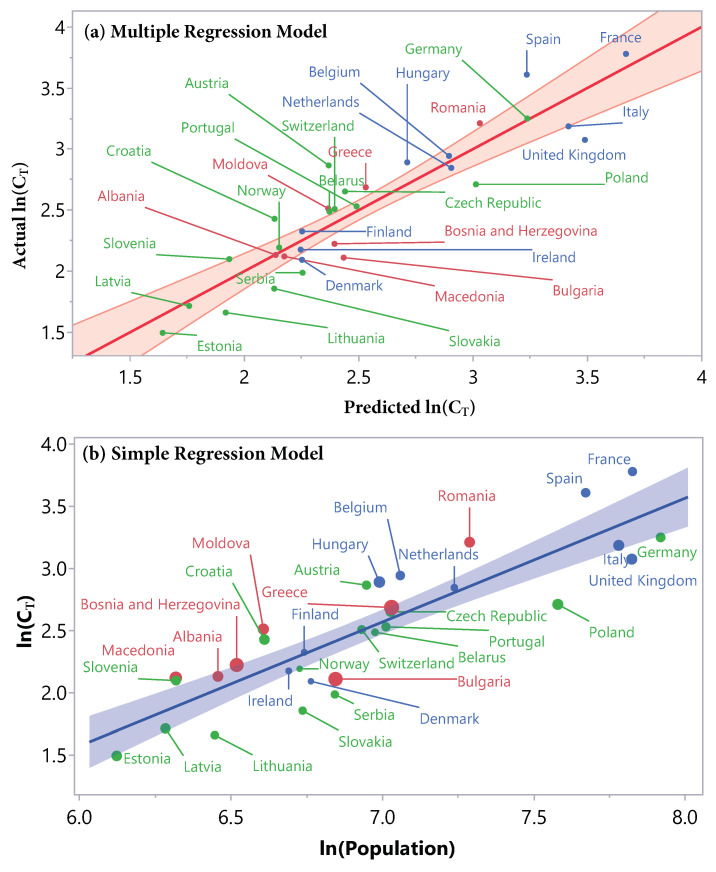
Graphical representations of the linear regression models for the logarithm of the threshold value $\ln(C_{T})$. (**a**) The multiple regression model ($R^{2}=85.0\%$, root mean squared error (RMSE) =0.23) contains three factors. The graph shows the predicted $\ln(C_{T})$ versus the actual $\ln(C_{T})$. The points roughly follow the diagonal line and are almost all inside the confidence region, showing a good model fit. For example, Germany is about four times bigger than Latvia, but both threshold values are very well predicted by the model. (**b**) The simple linear model ($R^{2}=77.2\%$, RMSE =0.27) with ln(Population) as the only factor shows the dominance of this factor. The size of points is proportional to the cCFR $\alpha_{2}$ of the second wave. In both graphs, the colors represent the corresponding cluster. There is no apparent structure with respect to point size or cluster, the linear relation is independent of these factors.

A graphical representation of the model is shown in Figure 3a. The predicted response value is displayed versus the actual response value. The better the points follow the diagonal line, the better the model fits the data. The results of the *t*-tests for every parameter in the model are displayed in Table 5.

For comparison, we also fitted a simple linear regression, with the logarithm of population size as only factor. This resulted in the following model:

(7) $$\ln\left(C_{T}\right)=-4.39+0.99\cdot \ln\left(P\right)$$

with $R^{2}$ dropping to $R^{2}=77.2\%$, and root mean squared error (RMSE) =0.27. The corresponding graph with the factor ln(P) on the *x*-axis and the response $\ln(C_{T})$ on the *y*-axis is shown in Figure 3b. The color of the points corresponds to the respective cluster, the size of the points is proportional to the cCFR $\alpha_{2}$ during the second wave.

**Figure 4:**
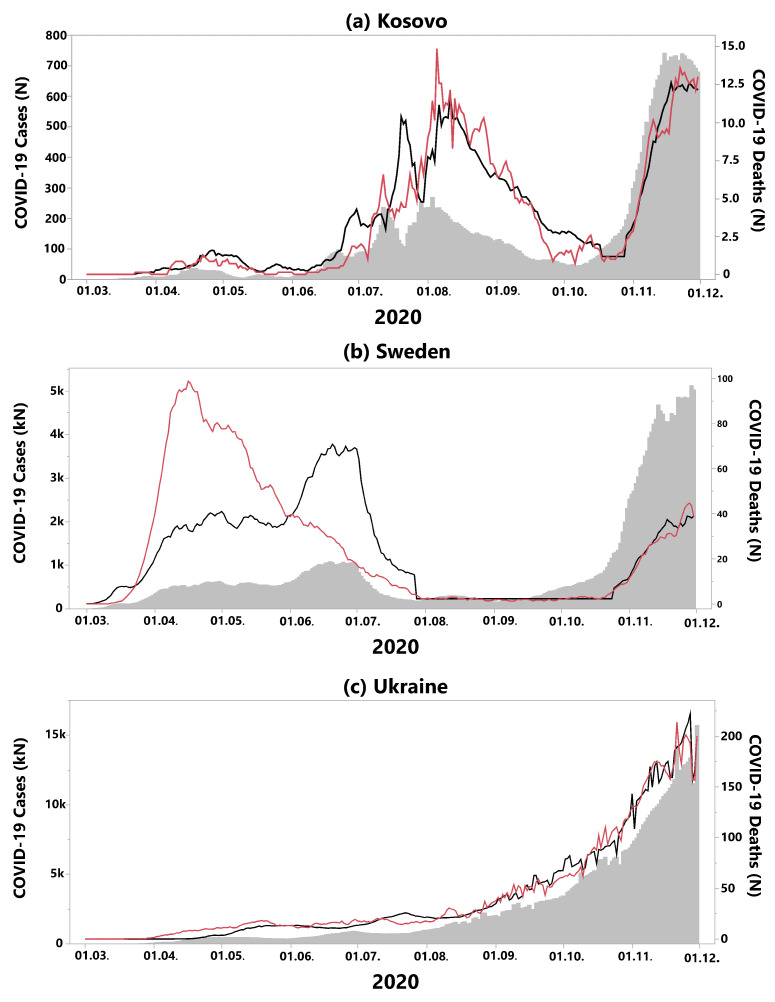
Countries where the three-phase-model was not applicable. (**a**) In Kosovo, the second phase with a relative low death rate was missing. Instead, the country suffered a second wave during summer, directly followed by a third wave. (**b**) In Sweden, at the beginning of the first wave, the recorded death rate was much higher than predicted by the incidences. In the second wave, the model fitted the data well, with a cCFR of $\alpha_{2}=0.8\%$. (**c**) In Ukraine, there were no waves; instead, COVID-19 incidences and related deaths increased constantly over the course of the year 2020.

Three European countries displayed exceptional courses of the pandemic; hence, our three-phase-model was not suited to describe the course of the pandemic (Figure 4):

- In Kosovo, the second phase with generally low death rates and defined by an uncoupling of incidences and death rates is missing (Figure 4a). Instead, the country suffered from a second wave during summer, directly followed by a third wave.
- It is well known that Sweden chose a different way to respond to the pandemic compared to neighbouring states in terms of restrictive measures [21]. In particular, during the first wave, the recorded death rate was much higher than predicted by the incidences, probably due to a relatively low level of political protective measures (Figure 4b). In the meantime, Sweden changed its policies with strict protective measures for the population [22]. Consequently, in the second wave, our model fits the data well, with a cCFR of $\alpha_{2}=0.8\%$.
- In Ukraine, no waves could be observed to date, instead COVID-19 incidences and related deaths increased constantly over the course of the year 2020 (Figure 4c).

**Figure 5:**
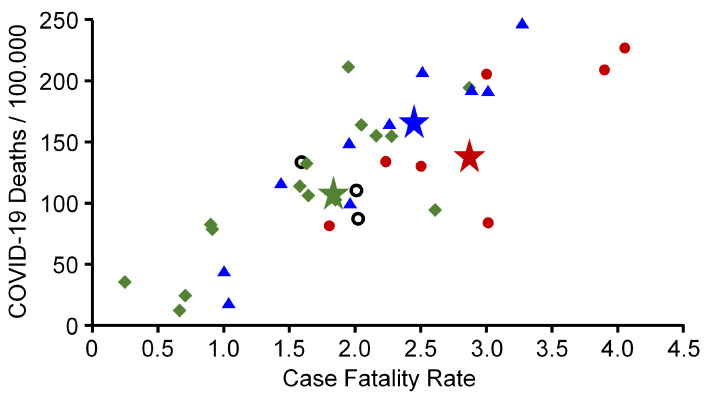
Cumulative COVID-19 associated death incidence per 100,000 inhabitants and cumulative case fatality rates of European countries with more than 1 million inhabitants. Stars (average) indicate the values for the total population of the corresponding cluster. Data were obtained on 13 April 2021 from the homepage of the World Health Organization [19]. Red circles, green squares, blue triangles = countries belonging to cluster 1, 2, and 3, respectively. Open black circles: countries belonging to no cluster.

The phenomenon of clustering due to similar epidemiological patterns raises the question of whether it is favourable for a country to belong to a certain cluster. Therefore, current data from the WHO were used to calculate the cumulative incidence of COVID-19 associated deaths and the cumulative CFR for each of the countries examined herein. Figure 5 shows that countries belonging to cluster 1 and 3 had similar COVID-19 associated death incidences and CFRs, although there was a high variance especially of countries belonging to cluster 3. When calculating both values for the total population of all countries belonging to a cluster, cumulative incidence of COVID-19 associated deaths were 138.2, 108.1, and 166.3 for cluster 1, 2, and 3, respectively. The CFR of cluster 1, 2, and 3 was 2.9%, 1.8%, and 2.5%, respectively (stars in Figure 5). This finding suggests that, in summary, it was probably favourable for a country to have been affected by a moderate first wave (cluster 2) while it was unfavourable to miss out on the first wave (cluster 1), or to be struck by a disastrous first wave (cluster 3)—although Finland and Denmark, both belonging to cluster 3, exhibited a cumulative low COVID-19 burden. In Denmark, 42.08 of 100,000 individuals died from COVID-19 (CFR 1.01%) and in Finland 15.84 of 100,000 inhabitants (CFR 1.03%). The highest death incidence was noticed in Hungary (245.37/100,000 inhabitants) (CFR 3.29%) and in Bosnia and Herzegovina (226.81/100,000 inhabitants) (CFR 4.05%) belonging to cluster 3 and cluster 1, respectively.

## 5. Discussion

Our data show that COVID-19 has spread inhomogeneously across Europe during the first and second waves. We conducted a time series analysis, which showed that our three-phase-model (first wave–in-between waves–second wave) describes the course of the pandemic very well in 32 out of 35 European countries.

The coupling of COVID-19 incidences and death rates could be shown in two distinct phases, namely the first and second waves in the spring and fall of 2020, whereas an uncoupling could be found in the phase in between waves. Once a certain individual threshold of infection rates was reached, death rates and incidences started to re-couple and deaths started to increase. This finding is important as health care systems across Europe vary greatly and display fundamental differences in ICU capacities and medical resources. If the individual coupling threshold in certain countries could be predicted, preparations for distribution of medical resources and staff could be undertaken early and an overcrowding of hospitals as well as overflowing ICUs might be limited. We have previously shown that individual coupling thresholds can be successfully applied to smaller geographical regions within a country to further maximize preparation efforts as well as political measures for closures of schools and businesses [3]. These individual thresholds for countries as well as defined geographical regions could define the way countries and communities handle lockdown measures and guide school opening strategies as well as economy stabilizing business opening concepts.

In addition, we defined so-called “shift parameters” that represent the number of days between reported infections and reported COVID-19 associated deaths. The shift factors show a wide range in the two waves (phase 1 and phase 3) across Europe. The wide range of values emphasises that it is crucial to account for these shifts in order to obtain reliable cCFRs for individual countries. The shift parameters increased from nine days in the first wave to 12 days in the second wave across Europe. The increase in time between infection and death could possibly be explained by a certain immunity that had occurred by the second wave resulting in a less vulnerable population and a lower overall CFR in the second wave. The cCFRs in the second wave ranged from 0.5% in Denmark to 4.2% in Greece. Overall, these values are significantly lower than in the first wave. Possible explanations include increased test rates, protection of the most vulnerable individuals within a population and other political measures. In addition, medical knowledge of the virus and its resulting disease had improved from wave one to wave two, adding more specific pharmacological treatments as well as progress in hospital organization and resource management.

We postulate that there are two additional effects that lead to lower cCFRs in the second wave. First, the most vulnerable individuals within a population had already been affected during the first wave, and outcomes were detrimental. Second, there is a large body of evidence showing a high number of unreported, often asymptomatic cases of SARS-CoV-2 infections [23,24] that might have contributed to a certain level of immunity across European countries with limited means for widespread testing. A recent meta-analysis of seroprevalence estimates a global factor between confirmed and actual infections of 11.9 on national levels [25], pointing towards a high level of undetected infections. Hence, the exposure to the virus had been much higher during the first wave than reported by the registered positive tests, which might have led to a partial immunity of the exposed population. Partial immunity of the population would also explain the observation that, after one year of the pandemic, countries belonging to cluster 2 in general had lower cumulative CFRs and lower cumulative COVID-19 associated death incidences than countries belonging to cluster 1 (Figure 5).

Adding both effects might have lowered the overall vulnerability of the population in the second wave and hence might have contributed to the lower cCFRs reported in our data. If these statements were true, we should also have observed the opposite: countries that were mildly affected in the first wave would have suffered from higher cCFRs in the second wave. These switches from “high to low” and from “low to high” respectively are called a “flip effect”.

Indeed, we could show that the geographical spread of SARS-CoV-2 in Europe followed a distinct pattern with similarities and discrepancies in death rates over time. Based on those similarities, we were able to define geographical clusters of countries showing a similar dynamic as the pandemic evolved during the first and second waves in 2020.

In summary, the western part of Europe (cluster 3) went from high cCFRs to low cCFRs, while the countries in the southeastern parts of Europe (cluster 1) exhibited the opposite starting off with low CFRs in the first wave and flipping to extremely high CFRs in the second wave, adding evidence to the “flip effect.” A particularly clear view of this flip effect is offered by the rank parallel plot in Figure 2b. While the red lines (cluster 1) are all trending upwards, the blue lines (cluster 3) are trending downwards. Without the presence of the flip effect, the lines in this diagram would be mostly horizontal. The situation in cluster 2 is similar to cluster 1 but less pronounced, with an average rank loss of −3.8, compared to −13.6 in cluster 1. In cluster 1, the average duration of the first wave was 138.4 days, compared to 112.5 days in cluster 3, a difference of 26 days. The countries in cluster 3 were much more affected by the first wave, potentially leading to more drastic restriction measures and hence a faster decline of incidences than in less severely affected countries.

It is an interesting result by itself that, although the clustering algorithm did not use any geographical information, the clusters are clearly assignable to different regions of Europe (see Figure 2a). The first cluster is entirely located in the southeastern part of Europe, with Greece, Romania and Bulgaria being its three largest representatives. As mentioned, the southeastern part of Europe was less affected by the first wave, but heavily affected by the second wave. Cluster 3, showing the opposite behaviour, covers the whole western part of Europe, from Denmark to Italy, with France, UK, and Italy being its three largest representatives. It is worth noting two exceptions: on one hand, Portugal was warned by the terrible course of the pandemic in its only neighbouring state Spain, and therefore imposed lockdown measures at a very early stage [26], keeping the incidences and the death rate relatively low during the first wave ($\alpha_{1}=4.3\%$). On the other hand, Hungary had low incidence numbers, but an unusually high cCFR of $\alpha_{1}=13.7\%$. At least partially, this might be explained by a low test rate in Hungary during these months [27].

The questions arises as to why certain geographical regions in Europe follow certain similar patterns of viral spread and CFRs. Recent data point towards a link between national gross domestic product (GDP), tourism and COVID-19 cases during the initial phase of the pandemic. Countries with a high GDP as well as high tourist activity had more COVID-19 cases during the initial first wave than countries with a lower GDP and lower tourist activity [28]. These findings support our data and are in keeping with our three geographical clusters. Clusters 2 and 3 representing the Central and Western parts of Europe, with higher GDPs on average as well as high levels of tourism as well as business travel facilitating the initial spread. Cluster 1 representing the southeastern parts of Europe with a relatively low average GDP suffered tremendously from the second wave after it had been spared from the first wave due to low tourist and business travel activity. Countries with higher GDP such as clusters 1 and 2 were less affected by the second wave as health care systems were able to adapt, and medical innovations could be widely applied and resources distributed.

Interestingly, the weather seems to have an impact on viral spread and COVID-19 [29,30]. Data from Spain, which is part of our “Western” cluster 3, show that UV-radiation during the winter months of 2019–2020 seemed to be inversely correlated with the number in infections in the spring of 2020. It remains unclear whether this effect is caused by direct effects of UV-radiation on virus replication or whether it is an effect of increased immunity by higher Vitamin-D levels [31]. The weather-hypothesis has been studied elsewhere. Incidences were much higher in the North of Italy for example than in southern Italian regions. Again, UV-radiation from North to South correlated inversely with COVID-19 [32]. Our cluster 3 is mainly composed of “southwestern” European countries defined by high UV-radiation and a dry southern climate which might partly explain the relatively mild second wave with low CFRs.

We already elucidated how the fit of our three-phase-model implies the existence of a threshold value for each country individually at the beginning of the second wave. As long as the incidences stayed below this threshold value during the summer months, changes in COVID-19 incidences did not affect the number of related deaths in the same way as during the first and second wave.

The aim of the multiple regression model was to find out if it is possible to predict the threshold value, where the COVID-19 incidences and the death rates re-couple leading to increasing death rates and marking the beginning of a new wave. The multiple regression model has an $R^{2}$ value of $R^{2}=85.0\%$, which means that the model can explain 85% of the variation in the data (see Figure 3a). The comparison with the one-dimensional model (see Figure 3b) that exhibited an $R^{2}$ value of $R^{2}=77.2\%$ proves two statements: first, the logarithm of the population (ln(P)) is the dominant factor for the prediction of the threshold. Second, adding the factors $\alpha_{1}$ and $p_{2rel}$ improves the model. This is also reflected by the significant effect tests (see Table 4). The fact that the parameter estimates for ln(P) are close to 1 (0.99 in the simple regression, 0.86 in the multiple regression) is highly plausible and implies the following interpretation: a 10-fold increase in population size leads to a $\left(10^{0.86}\right)$7.24-fold increase in the threshold value. The two other factors in the multiple regression model, $\alpha_{1}$ and $p_{2rel}$, can be interpreted as measurements of the severity of the pandemic before the second wave. A positive sign of these factors indicates the degree of preparedness: a more extended first wave, and a summer with slightly higher incidences, always staying under the predicted coupling threshold, might have produced higher rates of immunity within communities, potentially decreasing the severity of the following second wave and delaying the re-coupling of incidences and rising death rates.

We can draw another conclusion from Figure 3b by looking at the size and color of the points. The color represents the cluster, the size corresponds to the cCFR $\alpha_{2}$. There is no structure visible with respect to the color or size of the points. This means that the linear connection expressed by the regression model for the prediction of the threshold is independent of the other factors. It holds through all clusters and for any severity of the second wave, expressed by $\alpha_{2}$.

Our study has limitations. Our analyses are based on observational data for 35 European countries provided by the ECDC. The definition of a COVID-19 related death might vary from country to country, as might the reliability of the reporting itself. In addition, it is important to keep in mind that we analyse case fatality rates and not the mortality of the novel coronavirus. Due to the high number of unreported cases, the cCFR are an overestimation of mortality. The analysis of excess mortality might provide additional information about the threat that COVID-19 poses for the population [5,33,34]. The testing strategies vary a lot from country to country, and changed within each country over the course of the pandemic. This introduces a source of variation to the incidence data that we did not control for.

In addition, our predicted thresholds are estimated ex-post. Further investigation is needed to assess the predictive power for future data. Another interesting aspect could be the analysis of possible interactions between, for instance, countries in the same cluster. Two other approaches could be used to study COVID-19 time series on a European level in further studies: entropy ratios [35] and a detrended cross-correlation analysis [36]. Finally, results of time series analysis might facilitate predicting the peak of the epidemic in terms of new cases or new deaths in each country [37].

Recently, mutations of the corona virus have been detected in many European countries. At this point, we cannot predict if our three-phase model could be adjusted to the seemingly more aggressive spread of newer virus mutations which seem to elucidate an entirely new dynamic in terms of infectiousness as well as aggressiveness of the resulting COVID-19.

## 6. Conclusions

Our results show that, in 32 out of 35 European countries, the COVID-19 pandemic can be accurately depicted by a three-phase-model. In the first and third phase, the corrected case fatality rates were coupled to the incidences. However, in the second phase, we see a random unconnected behaviour of incidences and deaths indicating an uncoupling of COVID-19 related deaths with incidences as long as the incidences stay below a specific threshold.

Three distinct clusters were obtained when clustering the parameters of the three-phase-model: The first cluster in the south east of Europe, with relatively low cCFRs in the first wave and high cCFRs in the second wave. A second cluster in the centre of Europe, with low cCFRs in the first wave, and slightly higher cCFRs in the second wave, and a third cluster in the western part of Europe, with very high cCFR during the first wave, and low cCFRs in the second wave.

Finally, a strong linear relationship was found for the prediction of the threshold value at the beginning of the second wave, where the incidences and the COVID-19 related death rates reconnected. Using the size of the population, the corrected case fatality rate from the first wave, and the relative death rate during the second phase, the model predicts a 7.24-fold increase of the threshold for a 10-fold increase of the population. Below this country-specific threshold, an increase in COVID-19 incidences did not lead to a significant rise of COVID-19 related deaths.

## Figures and Tables

**Table 1:** Results of the time series analysis for 35 European countries. The first 7 columns contain the parameters of the time series model (Equation 1): $\alpha_{1}$ and $\alpha_{2}$ = corrected case fatality rates of phase 1 and phase 3. $d_{1}$ and $d_{2}$ = shift parameters (days) of phase 1 and 3. Date of switch between phase 1 and phase 2: March 1 $+t_{1}$ (days). Date of switch between phase 2 and phase 3: March 1 $+t_{2}$(days). $p_{2}$ = average deaths in phase 2. Δr = the difference of the α-ranks. $C_{T}$ = threshold value (Equation (3)). The last column contains the result of the cluster algorithm with three clusters. H. = Herzegovina.

Country	$\alpha_{1}$	$d_{1}$	$t_{1}$	$t_{2}$	$\alpha_{2}$	$d_{2}$	$p_{2}$	Δr	$C_{T}$	Cluster
Albania	3.2%	4	141	187	1.9%	3	4.173	−18	136.1	1
Bosnia and H.	5.4%	21	136	228	3.5%	10	7.892	−11	168.0	1
Bulgaria	4.1%	15	140	221	3.7%	14	7.125	−22	129.6	1
Greece	4.7%	8	164	252	4.2%	17	5.100	−17	487.0	1
Macedonia	4.9%	6	131	223	2.7%	8	4.545	−12	132.6	1
Moldova	4.4%	11	110	166	2.1%	11	7.642	−14	326.4	1
Romania	6.0%	10	147	221	1.9%	2	39.945	−1	1627.4	1
Austria	3.4%	13	97	220	1.3%	13	1.107	−3	736.1	2
Belarus	0.6%	1	108	237	0.8%	23	4.896	−4	308.0	2
Croatia	4.1%	18	137	196	1.8%	10	1.486	−10	269.1	2
Czech Rep.	3.4%	11	130	198	1.7%	10	1.460	−10	450.3	2
Estonia	3.5%	11	81	250	1.8%	20	0.059	−13	31.4	2
Germany	4.5%	14	117	227	1.5%	18	6.457	−1	1778.6	2
Latvia	2.6%	24	133	228	1.7%	10	0.109	−18	52.1	2
Lithuania	3.2%	8	114	212	1.1%	9	0.175	−2	46.0	2
Norway	2.9%	16	81	257	0.5%	18	0.303	4	156.6	2
Poland	5.8%	6	78	211	2.0%	11	11.327	−5	515.4	2
Portugal	4.3%	5	100	195	1.3%	6	4.024	0	340.4	2
Serbia	2.3%	1	190	231	1.0%	8	1.245	−5	97.4	2
Slovakia	2.0%	10	122	204	1.0%	17	0.145	−7	72.3	2
Slovenia	7.2%	13	112	225	1.5%	14	0.302	9	126.0	2
Switzerland	4.8%	10	103	227	1.2%	14	1.054	8	323.4	2
Belgium	16.9%	5	102	213	1.4%	15	4.643	19	879.7	3
Denmark	5.2%	1	108	198	0.5%	25	0.377	20	124.3	3
Finland	5.9%	15	85	257	0.6%	2	0.348	21	212.4	3
France	20.7%	7	97	220	1.3%	13	26.506	22	6011.3	3
Hungary	13.7%	8	168	202	2.2%	1	1.943	0	779.3	3
Ireland	6.8%	10	125	212	0.6%	19	0.716	23	150.7	3
Italy	14.3%	4	120	217	2.0%	11	12.429	5	1535.0	3
Netherlands	13.1%	4	97	206	0.8%	14	2.834	24	702.3	3
Spain	11.6%	5	93	177	1.4%	9	6.521	13	4064.4	3
UK	15.4%	2	130	203	2.0%	21	15.033	4	1190.1	3
Kosovo	Model not applicable						
Sweden	Model not applicable						
Ukraine	Model not applicable									

**Table 2:** The 5-point summary statistics of the five model parameters estimated for 32 European countries.

	$\alpha_{1}$	$d_{1}$	$\alpha_{2}$	$d_{2}$	$p_{2\mathrm{rel}}$
Minimum	0.006	1	0.005	1	0.003
First Quartile	0.034	5	0.010	9	0.009
Median	0.048	9	0.015	12	0.019
Third Quartile	0.071	13	0.020	17	0.046
Maximum	0.207	24	0.042	25	0.239

**Table 3:** COVID-19 incidences and related deaths in July and August 2020 for four countries. Although the incidences increased significantly, the related deaths did not. Below a certain threshold, incidences and deaths are not necessarily connected (“uncoupling” of incidences and deaths).

Country	Incidences	Deaths	Incidences	Deaths
	July	July	August	August
Austria	3343	15	6209	15
France	22,313	441	91,370	352
Germany	14,439	168	33,683	157
Denmark	974	10	2975	9

**Table 4:** Mean values per cluster. While cluster 1 shows a strong decrease of relative position, in cluster 3, the relative rank increases by almost the same amount. In cluster 2, the average rank decreases by 3.8 points.

Cluster	N	$\alpha_{1}$	$\alpha_{2}$	$p_{2\mathrm{rel}}$	Δr
1	7	0.0467	0.029	0.164	−13.6
2	15	0.0364	0.013	0.017	−3.8
3	10	0.1236	0.013	0.020	15.1

**Table 5:** Parameters of the multiple regression model ($R^{2}=85.0\%$) for the prediction of $\ln(C_{T})$. All parameters contribute significantly (p<0.05) to the prediction, as shown by the *p*-values.

Term	Estimate	Standard Error	t Ratio	*p*-Value
$c_{0}$	−3.78	0.70	−5.43	<0.0001
$c_{1}$	0.87	0.10	8.31	<0.0001
$c_{2}$	2.86	1.01	2.82	0.0086
$c_{3}$	1.50	0.60	2.51	0.0182

## Data Availability

The data presented in this study are openly available from the website of the European Centre for Disease Prevention and Control [1].
